# Computational modelling of the antimicrobial peptides Cruzioseptin-4 extracted from the frog *Cruziohyla calcarifer* and Pictuseptin-1 extracted from the frog *Boana picturata*

**DOI:** 10.1038/s41598-024-55171-w

**Published:** 2024-02-27

**Authors:** María José Rengifo-Lema, Carolina Proaño-Bolaños, Sebastián Cuesta, Lorena Meneses

**Affiliations:** 1 Escuela de Ciencias Químicas, Facultad de Ciencias Exactas y Naturales, Pontificia Universidad Católica del Ecuador, Av. 12 de Octubre 1076 y Roca, Quito, Ecuador; 2 Universidad Regional Amazónica Ikiam, km 7 vía Muyuna, Tena, Ecuador

**Keywords:** Antimicrobial peptides, *Boana picturata*, *Cruziohyla calcarifer*, Molecular docking, Physicochemical properties, Pathogens, Biochemistry, Chemistry

## Abstract

A computational study of the peptides Cruzioseptin-4 and Pictuseptin-1, identified in *Cruziohyla calcarifer* and *Boana picturata* respectively, has been carried out. The studies on Cruzioseptin-4 show that it is a cationic peptide with a chain of 23 amino acids that possess 52.17% of hydrophobic amino acids and a charge of + 1.2 at pH 7. Similarly, Pictuseptin-1 is a 22 amino acids peptide with a charge of + 3 at pH 7 and 45.45% of hydrophobic amino acids. Furthermore, the predominant secondary structure for both peptides is alpha-helical. The physicochemical properties were predicted using PepCalc and Bio-Synthesis; secondary structures using Jpred4 and PredictProtein; while molecular docking was performed using Autodock Vina. Geometry optimization of the peptides was done using the ONIOM hybrid method with the HF/6-31G basis set implemented in the Gaussian 09 program. Finally, the molecular docking study indicates that the viable mechanism of action for both peptides is through a targeted attack on the cell membrane of pathogens via electrostatic interactions with different membrane components, leading to cell lysis.

## Introduction

In recent years, a progressive increase in antimicrobial resistance (AMR) has been observed. The main drivers of AMR are overuse and misuse of antimicrobials^1^. According to statistical models in 2019 there were 4.95 million deaths associated with antibiotic resistance, with a trend of increasing mortality rate^2^. It is estimated that by the year 2050, AMR will be the leading cause of death in the world^3,4^.

There is an urge to develop new effective antimicrobials to tackle this problem. The design of antimicrobials that are not prone to the different resistance mechanisms that exist towards antibiotics is quite complicated, since bacteria present different types of antimicrobial resistance^5^. Antimicrobial peptides appear as a promising antimicrobial strategy due to broad spectrum of activity, especially in combination with antibiotics and emerging technologies as nanoparticles^6,7^.

AMPs are molecules composed of 5–40 amino acids generated in different organisms such as bacteria, plants, animals and humans. AMPs, in addition to possessing natural antimicrobial properties, they may have anticancer activities as some evidence suggest^8,9^. AMPs are molecules that have amphipathic and cationic properties which are the main drivers to its interaction with microbial membranes. AMPs that act against bacterial membranes may be less susceptible to resistance and may have potential as effective drugs, since they are isolated from animal cells, such as polymorphonuclear leukocytes, macrophages and cells. Unlike traditional antibiotics, which target specific cellular activities, AMPs have three advantages: (1) broad-spectrum activity against various microorganisms; (2) lower likelihood of bacterial resistance due to mechanisms of attack and functionality through innate immune systems; and (3) they preferentially interact with bacterial rather than mammalian cells, making them more potent against microorganisms without inducing significant toxicity^10^. AMPs share common features; these include a small size and a linear or cyclic structure. The linear structure comprises amphipathic α-helices, whereas the cyclic structure contains one or more disulfide bridges that form a β-sheet^11^.

The structure of AMPs is composed of hydrophobic and hydrophilic amino acids, with a positive net charge (in the range of + 2 and + 9). These molecules including Bombinins, Dermaseptins and Temporins can be effective against a wide variety of antibiotic-resistant microorganisms. Some studies indicate that they are effective against some pathogens such as* Escherichia coli*, *Staphylococcusaureus*, *Salmonella sp., Vibrio parahaemolyticus, Listeria monocytogenes,* among others^12,13^. There are a few mechanisms of action of cationic AMPs. However, in general, these peptides exhibit membrane binding activity through electrostatic interactions between positive regions of AMPs with negative regions of enzymes, leading to the formation of a cavity in cell membranes, resulting in membrane permeability and eventually leading to overflow of bacterial contents, lysis of the microbial body and cell death^14^.

One of the best sources of antimicrobial peptides is the amphibian skin^15^. Over the years, some antimicrobial peptides have been identified in the skin of different amphibian species. For example, Dermatoxin, Dermaseptin, Plasticin, Phyloseptin and Cruzioseptin are peptides extracted from some amphibian species' skin^16–18^. Cruzioseptin (CZS) are antimicrobial peptides named after *Cruziohyla.* CZS were characterized from the skin secretions of *Cruziohyla calcarifer,* a frog distributed from northwestern Ecuador to the southeastern of Nicaragua^19^. Cruzioseptins are cationic peptides containing between 20 and 30 amino acid^17^. In vitro assays and computational methods have confirmed that Cruzioseptins-1, 2, and 3 show antimicrobial activity against Gram-positive bacteria *S. aureus* and Gram-negative bacteria *E. coli* and the yeast *Candida albicans*^17,20^. Same as Cruzioseptins, Pictuseptins are antimicrobial peptides named after *Boana picturata,* a frog distributed in the northwest Ecuador and southwest of Colombia^18,21^. Cruzioseptins and Pictuseptins are relatively new molecules, which has aroused researchers' interest in determining its antimicrobial potential.

In this study, different computational methods are used to characterize Cruzoseptin-4 and Pictuseptin-1 concerning their physicochemical properties, secondary structure and mechanism of action based on molecular docking with enzymes and molecules present in the cell membrane of *S. aureus*, *E. coli* and *C. albicans*.

## Experimental

Cruzioseptin-4 and Pictuseptin-1 peptides were described from *Cruziohyla calcarifer* and *Boana picturata* frogs in previous studies^17,18^. We characterised these peptides in terms of their physicochemical properties an also perform molecular coupling of the peptides with enzymes and molecules present in the cell membrane of *S. aureus*, *E. coli* and *C. albicans* was performed. The antimicrobial assays with cruzioseptin-4 do not form part of this investigation as they are under publication process.

Different computational resources and methods were used in this study, such as web servers (Bio-Synthesis, Jpred4 and PepCalc)^22,24^, databases (Protein Data Bank)^25,26^, molecular visualization tools (PyMol and AutodockTools)^27,28^ and software for quantum mechanics calculations (Gaussian 09)^29^ and molecular docking (Autodock Vina)^30^.

### Physicochemical properties

PepCalc and Bio-Synthesis web servers were used to determine the physicochemical properties of the peptides, such as isoelectric point, hydrophobicity, hydrophilicity, number of positively and negatively charged amino acids, percentage of neutral, acid, basic, and hydrophobic amino acids, and net charge at pH 7^22,23^.

### Secondary structure prediction

The web servers Bio-Synthesis^23^, PredictProtein^22^ and Jpred4^24^ were used for peptide secondary structure prediction. Three servers were used to compare results and have a better degree of confidence in the results obtained. Although all three servers produce similar results, we found Jpred is the one that present more consistent results and therefore was the preferred one used.

### Computational molecular modelling

Cruzioseptin-4 and Pictuseptin-1 peptide structures were modelled using the builder GUI menu available in Pymol^27^. The Basic Local Alignment Search Tool (BLAST) was used to find similar sequences in the NCBI database. This tool compares the similarity based on the amino acid sequence and produce a score. This tool helps identify the same peptide in a different organism or similar peptides that can belong to the same family.

### Geometric optimization

Optimization of the three-dimensional structures of each peptide and molecules present in the cell membrane of *E. coli*, *S. aureus* and *C. albicans* was performed using Gaussian 09 software^29^. The ONIOM hybrid method with the HF/6-31-G basis set was used in this process which have been previously described and successfully used to describe properties and optimize peptides systems^31–33^.

The ONIOM method is an extrapolative scheme, it is considered a hybrid two-layer QM (Quantum Mechanics)/MM (Classical Molecular Mechanics) method, and it can also uniquely combine different methods, and can be easily extended to multiple layers. This method is applied to obtain a reliable relative energy difference between structures, which are chemically and physically more relevant than the absolute energy of the system. ONIOM have correct dimensionality and can be used directly for geometry optimization (as performed in this work), normal modes of vibration, frequency calculations and molecular dynamics simulations^34^. Geometry optimization is a method for predicting the three-dimensional arrangement of the atoms of a molecule by energy minimization^35^.

### Obtaining and cleaning of enzymes present in the cell membrane of pathogens

The enzymes in this study were obtained from the RCSB Protein Data Bank database^26^. All the preparation for docking studies was performed using PyMol software^27^.

### Molecular docking

For molecular docking calculations, utodockTools was used to prepare the ligands and proteins^28^. For the ligands, the optimized structures of the two peptides were chosen while for the proteins Penicillin-binding protein g-Acyl 2A (PDB:), AmiA hydrolase (PDB:), DNA gyrase B (PDB:), Penicillin Binding Protein Transglycosidase 1b (PDB:), Exo-B-(1,3)-glucanase (PDB:), and Secreted aspartic protease (PDB:) were selected. A standard flexibility of the peptide was allowed (total flexibility in the side chains and restricted in the amide bond), a spacing of 1 Å was chosen, and the grid restricted to the active site of each target. In the second stage of the docking, eleven molecules present in the cell membrane were modelled and the peptides chosen as receptors. Full flexibility of the ligands were allowed and the calculation grid was set to enclose the whole peptide. Spacing was also set to 1 Å. All calculations were performed with Autodock Vina^30^ which uses a distinctive set of optimization algorithms i.e. Broyden-Fletcher-Goldfarb-Shanno, particle swarm optimization, genetic algorithm, and others plus and a conformation-dependent scoring functions described elsewhere^36^.

## Results and discussion

### Physicochemical properties

The amino acid sequence of the peptides Cruzioseptin-4 and Pictuseptin-1 was dilucidated previously by molecular cloning and tandem mass spectrometry^17,18^. These two peptides were chosen as studies have demonstrated the antimicrobial activity of cruzioseptins and pictuseptins against some pathogens of interest^18^. Obtaining the physicochemical properties of peptides are very useful to characterize the molecule, group them into families and create the first hypothesis into their functions and mechanism of action. Therefore, with additional studies, this information will allow the creation of AMP-based drug design tools^37^. Table 1 shows the data of some parameters evaluated for the two peptides Cruzioseptin-4 and Pictuseptin-1.

**Table 1:** Physicochemical properties of Cruzioseptin-4 and Pictuseptin-1.

Parameter	Cruzioseptin-4	Pictuseptin-1
Amino acid sequence	GFLDVIKHVGKAALSVVSHLINE-NH_2_	GFLDTLKNIGKTVGRIALNVLT- NH_2_
Length	23	22
Molecular weight (g/mol)	2446.04	2342.96
Isoelectric point	6.92	9.99
Formula	C_112_H_185_N_31_O_30_	C_106_H_184_N_30_O_29_
Number of atoms	358	349
Negatively charged amino acids	2	1
Positively charged amino acids	2	3
Charge at pH 7	1.2	2
Hydrophobicity	0.522	0.489
Sequence composition in percent	Neutral: 21.74%	Neutral: 36.36%
	Basic: 17.39%	Basic: 13.64%
	Acid: 8.7%	Acid: 4.55%
	Hydrophobic: 52.17%	Hydrophobic: 45.45%

In Table 1, for Cruzioseptin-4, the charge at pH7 is 1.2. CZS-4 has two positively charged amino acids, two negatively charged amino acids, and an amide group at its C-terminal side which gives this peptide a basic character. It is also observed that half of the amino acids present a hydrophobic character (52.17%). Likewise, the predicted physicochemical properties of Pictuseptin-1 are shown in Table 1, where its sequence and length differ slightly of Cruzioseptin-4. The charge at pH7 is 3, and it has one negatively charged amino acid, three positively charged amino acids, and an amide group at its C-terminal side giving it a basic character. It is also observed that almost half of the amino acids have a hydrophobic character (45.45%).

Determining the charge of cationic peptides is a fundamental aspect, as it allows to predict the degree of antimicrobial activity. However, it should be considered that with positive charges higher than + 9, there is a risk of null or poor antimicrobial activity^38^. Some studies show that highly charged peptides are more toxic and have higher hemolytic activity. In addition, it has been shown that there is a correlation between hemolytic activity and the net charge of the peptide. A high positive charge translates into high hemolytic activity; it may affect the biological activity of antimicrobial peptides. Therefore, the peptides studied in this work could be expected to have low hemolytic activity (low toxicity)^39^. The high positive charge is characteristic of cationic peptides. However, their structure must also be taken into account, as this factor helps to determine their affinity for the cell membranes of the bacteria or pathogens of interest^40^.

The amino acid composition may help to predict secondary structures. Antimicrobial peptides (AMPs) composed of charged, hydrophobic and aromatic amino acids have the tendency, in most cases, to self-assemble and form alpha-helix-like structures^41^. Hydrophobicity is a parameter that correlates with haemolytic activity and determines the interaction of peptides with membranes and their mechanism of action. Peptides with high hydrophobicity tend to have low toxicity, so the peptides studied are expected to have this characteristic^39,42^. Linear AMPs are characterized by abundant hydrophobic amino acids such as Isoleucine, Glycine, Valine, Alanine, Phenylalanine and Leucine. It should be noted that Histidine is hydrophilic and, like Phenylalanine, is considered aromatic^37^. The presence of several hydrophobic amino acids explains the high value in this parameter concerning the sequence composition of Cruzioseptin-4 and Pictuseptin-1^37^.

The percentage of acidic composition of the peptide sequence is due to negative side-chain amino acids such as Aspartic Acid and Glutamic Acid. On the other hand, the basic percentage of the peptide sequence is due to the presence of positive side-chain amino acids such as Lysine, Arginine and Histidine^43^. It should be noted that for both Cruzioseptin-4 and Pictuseptin-1, the second majority composition in their sequence corresponds to the presence of neutral type amino acids (apolar, aromatic and polar) such as Glycine, Phenylalanine, Leucine, Threonine, Asparagine, Isoleucine, Valine, Alanine and Serine^44,45^.

The peptides related to Cruzioseptin-4 and Pictuseptin-1 were compared with other peptides to determine their similarity. Each sequence was analysed in the NCBI database, where Cruzioseptin-4 showed 89.96% similarity to Cruzioseptin-2 (accession number A0A193H362.1), 73.91% similarity to Cruzioseptin-3, 16 and 17 all from *C. calcarifer* (accession number A0A193H2X2X2X2.1, QVE54761.1, QVE54760.1).

Pictuseptin-1, showed 95.45% similarity to Pictuseptin-2 (accession number UNO36941.1), 90.91% similarity to Pictuseptin-4 (accession number UNO36943.1), 76.92% similarity with Pictuseptin-3 all from *B. picturata* (accession number UNO36942.1), 63.16% similarity with Cruzioseptin-12 from *C. calcarifer* (accession number A0A193H397.1), 66.67% similarity with Rsp-1 from B. *raniceps* (accession number P86187.1).

The identity study of the two peptides showed that they belong to the Cruzioseptins and Pictuseptins families, respectively. Regarding the similarity of Pictuseptin-1 with Rsp-1 of *B. raniceps*, it is interesting given that this amphibian species is not found in Ecuador but in neighbouring countries such as Argentina, Bolivia, Brazil, and Paraguay; it would be interesting to investigate these similarities^48,49^.

In addition, this analysis allowed us to verify that the results of the study of the physicochemical properties of the Cruzioseptin-4 and Pictuseptin-1 peptides carried out in the different web servers are congruent with the results already obtained for the different peptides^17,20,46,47^. It can be observed in Table 2 that the positive, negative and neutral charges are in similar positions. In addition, it can be seen that all peptides have a high percentage of hydrophobicity and neutral charges.

**Table 2:**
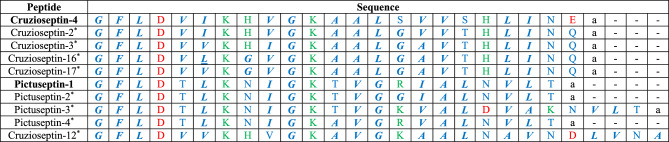
The sequence of peptides of the Cruzioseptin and Pictuseptin families.

Red letters: negative charges; green letters: positive charges; blue letters: neutral charges; Bold and italic letters: hydrophobic amino acids; a: amidation.

*Information taken by previous studies^17,20,46,47^.

Table 3 shows the hydrophobicity values of some peptides. For example, the highest hydrophobicity value corresponds to Cruzioseptin-2, followed by Cruzioseptin-3 and then the studied peptide Cruzioseptin-4. On the other hand, Pictuseptin-1 has the lowest hydrophobicity value compared to the peptides in Table 3. The variation in the hydrophobicity percentage values is due to the sequence of amino acids that each peptide possesses, as can be seen in Table 3 that there is enough similarity in the sequences between the peptides; however, in certain positions of the chains of these molecules, the type of amino acid varies. This explains why all these peptides have somewhat similar biological activity^15^.

**Table 3:** Physicochemical properties of five Cruzioseptins and one Pictuseptin.

Peptide	#aa	Alpha hélix [%]	Hydrophobicity [%]
Cruzioseptin-4	23	87.0	52.2
Cruzioseptin-2	23	73.9*	56.3*
Cruzioseptin-3	23	91.3*	52.3*
Cruzioseptin-16	23	82.6*	51.3*
Cruzioseptin-17	23	82.6*	49.2*
Pictuseptin-1	22	81.8	48.9

*Information taken by previous studies^17,46,47^.

A minimum inhibitory concentration (MIC) study was performed for two peptides, Cruzioseptin-16 (CZS-16) and Cruzioseptin-17 (CZS-17), where CZS-16 showed a haemolytic activity of 6.3–9.7%, while CZS-17 showed 0.7–11.6%. Therefore, both peptides were considered non-haemolytic^20^. Furthermore, it is observed in Table 2 that half of the amino acids for Cruzioseptins-16 and 17 are hydrophobic in character. Thus, it would be expected that the remaining peptides would exhibit similar haemolytic activity and level of toxicity.

### Secondary structure prediction

Secondary structures, such as alpha-helical and turns, are essential for determining the functionality of antimicrobial peptides and their interaction with cell membranes^50^. The alpha-helical structure is the most common motif in antimicrobial peptides, allowing them to insert into cell membranes. Turns can also improve the selectivity of peptides, although there are not many studies on this type of structure^51^.

Table 4 shows the results obtained for the predominant secondary structures (alpha helix or turns) of the studied peptides.

**Table 4:** Secondary structure of Cruzioseptin-4 and Pictuseptin-1 predicted by JPred4 and PredictProtein.

Web server	Predicted secondary structure for cruzioseptin-4 (bold) and pictuseptin-1 (italics)																							#aa	Helix alpha [%]	Turns [%]
JPred4	**t**	**h**	**h**	**h**	**h**	**h**	**h**	**h**	**h**	**h**	**h**	**h**	**h**	**h**	**h**	**h**	**h**	**h**	**h**	**h**	**h**	**t**	**t**	23	87.0	13.0
	**G**	**F**	**L**	**D**	**V**	**I**	**K**	**H**	**V**	**G**	**K**	**A**	**A**	**L**	**S**	**V**	**V**	**S**	**H**	**L**	**I**	**N**	**E**	–NH_2_		
	*t*	*t*	*h*	*h*	*h*	*h*	*h*	*h*	*h*	*h*	*h*	*h*	*h*	*h*	*h*	*h*	*h*	*h*	*h*	*h*	*t*	*t*		22	81.8	18.2
	*G*	*F*	*L*	*D*	*T*	*L*	*K*	*N*	*I*	*G*	*K*	*T*	*V*	*G*	*R*	*I*	*A*	*L*	*N*	*V*	*L*	*T*		–NH_2_		
Predict protein	**t**	**h**	**h**	**h**	**h**	**h**	**h**	**h**	**h**	**h**	**h**	**h**	**h**	**h**	**h**	**h**	**h**	**h**	**h**	**h**	**h**	**h**	**t**	23	91.3	8.7
	**G**	**F**	**L**	**D**	**V**	**I**	**K**	**H**	**V**	**G**	**K**	**A**	**A**	**L**	**S**	**V**	**V**	**S**	**H**	**L**	**I**	**N**	**E**	–NH_2_		
	*t*	*h*	*h*	*h*	*h*	*h*	*h*	*h*	*h*	*h*	*h*	*h*	*h*	*h*	*h*	*h*	*h*	*h*	*h*	*h*	*t*	*t*		22	86.4	13.6
	*G*	*F*	*L*	*D*	*T*	*L*	*K*	*N*	*I*	*G*	*K*	*T*	*V*	*G*	*R*	*I*	*A*	*L*	*N*	*V*	*L*	*T*		–NH_2_		

*h* helix alpha, *t* turns.

The secondary structure predictions for Cruzioseptin-4 shows that an alpha-helical structure prevails and turns are also present in some regions of the peptide. According to the Jpred4 web server, the percentage of alpha-helices is 87.0%, and of turns is 13.0%. Compared to the webserver PredictProtein, the percentage of alpha-helices is higher, being 91.3%, and thus, the rate of turns will be lower with a presence of 8.7%^21,22^. For Pictuseptin-1, web servers show that an alpha-helix structure predominates and that there are turns in some peptide regions. According to the Jpred4 web server, the percentage of alpha-helices is 81.8%, and the percentage of turns is 18.2%. Compared to the PredictProtein web server, the percentage of alpha-helices is higher, at 86.4%, and therefore the percentage of turns is lower, at 13.6%^24,25^.

When comparing the results with those of Cruzioseptin-4, it can be seen that Pictuseptin-1 has a lower number of regions with alpha-helix structures and therefore has a higher number of regions with turns structures. This would be influenced by the composition of the sequence, i.e., by the presence of hydrophobic, aromatic and charged amino acids, given that cruzioseptin-4 is composed of a higher number of amino acids with these characteristics (Table 1)^37^.

The difference in the results between the two web servers Jpred4 and PredictProtein for Cruzioseptin-4 occurs in the result of the amino acid Asparagine (N), where, according to JPred4, the structure is of the -turns type (t), and according to PredictProtein the structure is of the alpha-helix type (h). On the other hand, for Pictuseptin-1, the difference lies in the amino acid Phenylalanine (F), where, according to JPred4, the structure is of the -turns (t) type, and according to PredictProtein, the structure is of the alpha-helix (h) type^21,22,24,25^.

Jpred4 is known to provide predictions using the JNet algorithm and makes predictions about solvent accessibility and rolled-up regions. Moreover, this web server has an accuracy of 81.5%^24^. The PredictProtein web server uses analytical methods to identify functional regions, protein–protein binding sites, protein-polynucleotide binding sites, point mutation effect predictions and homology-based inference of Gene Ontology terms. This web server is known to have an accuracy of 76%^24,25^. The difference in methods used by each program reflects the unequal results obtained concerning secondary structures. Jpred4 has one of the most accurate methods for the prediction of secondary structures and therefore offers results that are more accurate, thus, the results obtained through this web server have a higher reliability for further calculations.

**Figure 1:**
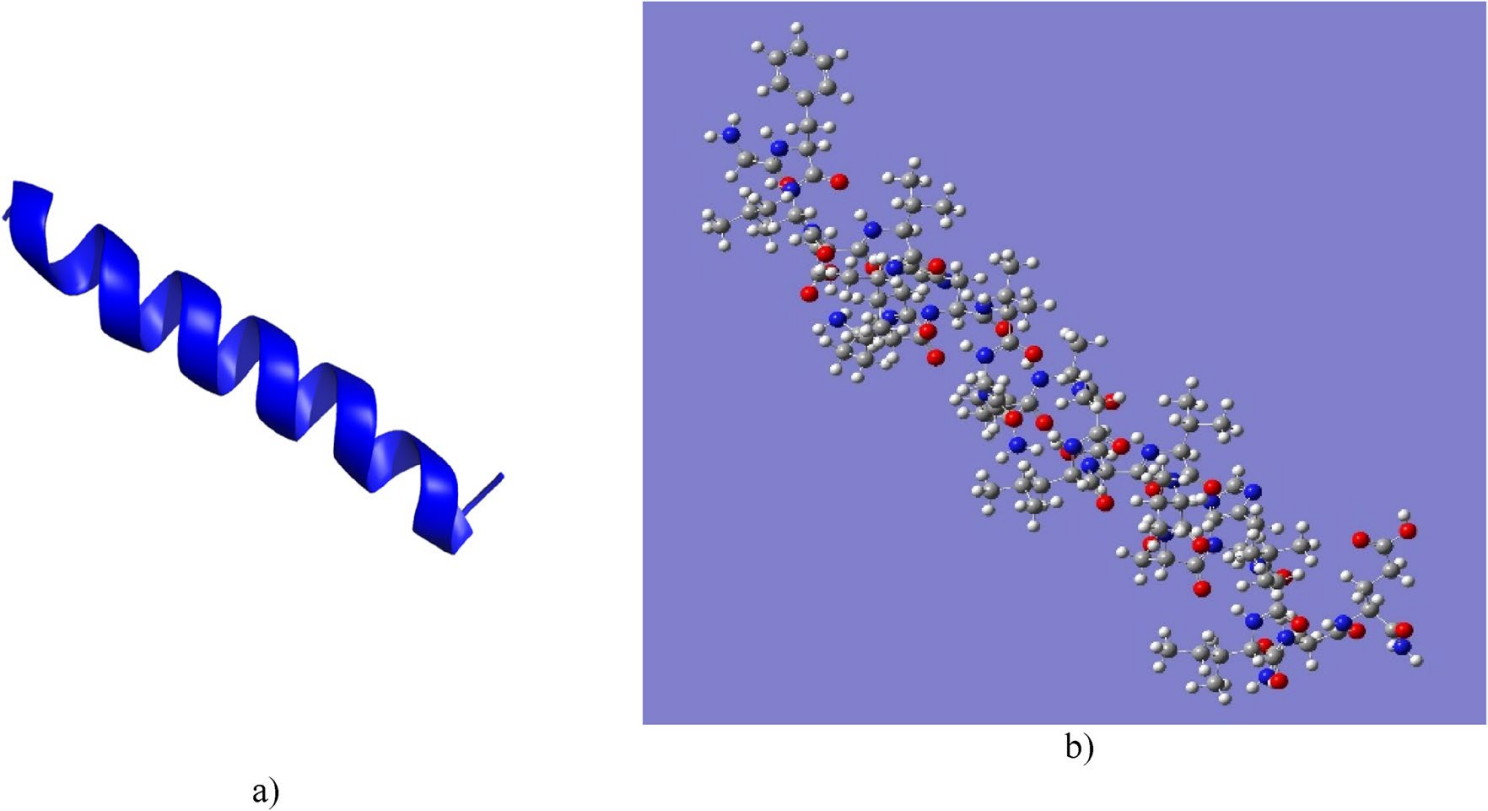
Three-dimensional structure models of the Cruzioseptin-4. (**a**) Model obtained by PyMol. (**b**) Model optimised by the ONIOM HF/ 6-31G method.

Figure 1a shows the three-dimensional structure of Cruzioseptin-4 obtained in PyMol, and Fig. 1b shows the optimised structure of the peptide in Gaussian 09, where the predominant secondary structure is an alpha helix, which is in agreement with the information obtained from the web servers^27,29^.

**Figure 2:**
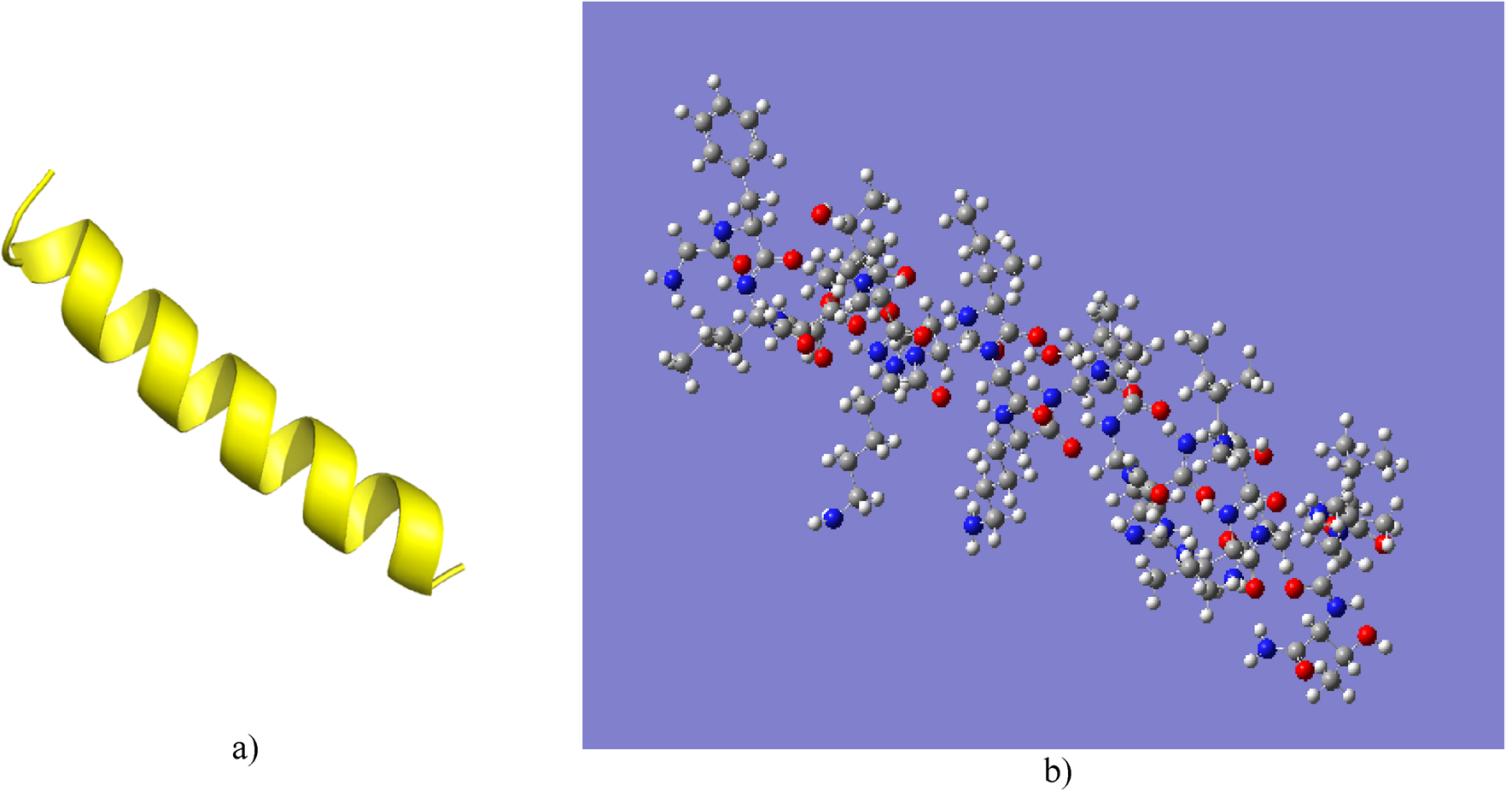
Three-dimensional structure models of the Pictuseptin-1. (**a**) Model obtained by PyMol. (**b**) Model optimised by the ONIOM HF/ 6-31G method.

Figure 2a shows the Pictuseptin-1 three-dimensional structure obtained in PyMol and 2b shows the optimised structure of the peptide in Gaussian 09, where it can be seen that the predominant secondary structure is alpha-helical^27,29^.

Furthermore, the results obtained from the web servers on the secondary structure predictions agree with the optimised structure of Pictuseptin-1 and Cruzioseptin-4, since the predominant conformation is alpha-helical. However, there is a more significant presence of turns in Pictuseptin-1 (Fig. 2a) than in Cruzioseptin-4 (Fig. 1a). In addition, the analysis of the identity and similarity of the peptides allowed us to verify that the study of the secondary structures performed in the web servers JPred4 and PredictProtein agree with the predominant alpha-helical structure for Pictuseptin-1 as for Cruzioseptin-4. The peptides with similar sequence and properties to those studied in Tables 2 and 3 have a predominantly alpha-helical structure^19,37^.

### Molecular docking

The molecular docking of Cruzioseptin-4 and Pictuseptin-1 with different enzymes of the cell membrane from *Staphylococcus aureus, Escherichia coli* and *Candida albicans* bacteria are shown in Table 5.

**Table 5:** Docking score of Cruzioseptin-4 and Pictuseptin-1 for different enzymes of the cell membrane from *Staphylococcus aureus*, *Escherichia coli* and *Candida albicans.*

Microorganism	Enzyme	Known inhibitor	Docking score (kcal/mol)		
			Known inhibitor	Cruzioseptin-4	Pictuseptin-1
*Staphylococcus aureus*	Penicillin-binding protein g-Acyl 2A	Ceftobiprole	− 9.5	− 6.2	− 7.1
	AmiA hydrolase	Muramyl tetrapeptide	− 7.1	− 6.0	− 6.2
*Escherichia coli*	DNA gyrase B	ADP	− 10.4	− 5.6	− 5.7
	Penicillin Binding Protein Transglycosidase 1b	Moenomycin	− 7.3	− 6.1	− 6.6
*Candida albicans*	Exo-B-(1,3)-glucanase	Castanospermine	− 7.0	− 5.1	− 5.9
	Secreted aspartic protease	A70*	− 7.7	− 6.3	− 5.5

*A70: N-etil-N-[(4-metilpiperazin-1-yl)carbonil]-D-fenilalanil-N-[(1S,2S,4R)-4-(butilcarbamoil)-1-(ciclohexilmetil)-2-hidroxi-5-metilhexil]-L-norleucinamida.

Table 5 shows the docking values expressed in kcal/mol of the different known enzymes and inhibitors with Cruzioseptin-4. It is observed that this peptide does not offer a higher docking score than that of its known inhibitor in all cases. However, the peptide has a higher docking score for Penicillin-binding protein g-acyl 2A, which is an enzyme present in the cell membrane of *Staphylococcus aureus* bacteria, and has a high-docking value for secreted aspartic protease, which is present in the cell membrane of yeast *Candida albicans*^52,53^.

Similarly, Table 5 shows the docking values of the different known enzymes and inhibitors with Pictuseptin-1, where it is observed that this peptide does not show a higher docking score than its known inhibitor in all cases. However, it can be observed that this peptide has a higher docking score with Penicillin g-Acyl 2A binding protein; likewise, it presents a high docking value with Penicillin Transglycosidase 1b binding protein, which is present in the cell membrane of *Escherichia coli* bacteria^54^.

Comparing the results obtained for the two peptides studied, it is observed that the docking values of Pictuseptin-1 with the enzymes studied are better than Cruzioseptin-4, because the peptide has lower values than Cruzioseptin-4, except the result obtained with the secreted aspartic protease present in the cell membrane of the yeast *Candida albicans* since Cruzioseptin-4 presents a more negative value of − 6.3 kcal/mol, in contrast with Pictuseptin-1, it is − 5.5 kcal/mol.

The mechanism of action of cationic peptides is understood to be through electrostatic interactions between the peptides and cell membranes of bacteria or pathogens^55^. In enzyme–substrate interactions, the charge and size of the peptide are essential^56^. Based on the results obtained in Table 1, which shows the charges of the peptides, it is observed that the highest charge corresponds to Pictuseptin-1 (+ 3); on the other hand, the lowest charge is Cruzioseptin-4 (+ 2). Concerning size, the number of atoms present in Cruzioseptin-4 is 358 atoms in its structure, and that of Pictuseptin-1 is 349 atoms. All these factors may explain why Pictuseptin-1 shows a better docking score with enzymes present in the cell membrane of the studied bacteria, as it has a better structure and size that allows it to interact better with the active sites of the enzymes.

The Penicillin-binding protein g-Acyl 2A is the key to β-lactam resistance in *Staphylococcus aureus* strains, as this enzyme provides transpeptidase activity that enables cell wall synthesis. In addition, this enzyme has a high molecular weight, N-terminal transmembrane anchorage and high-spectrum resistance^57^.

**Figure 3:**
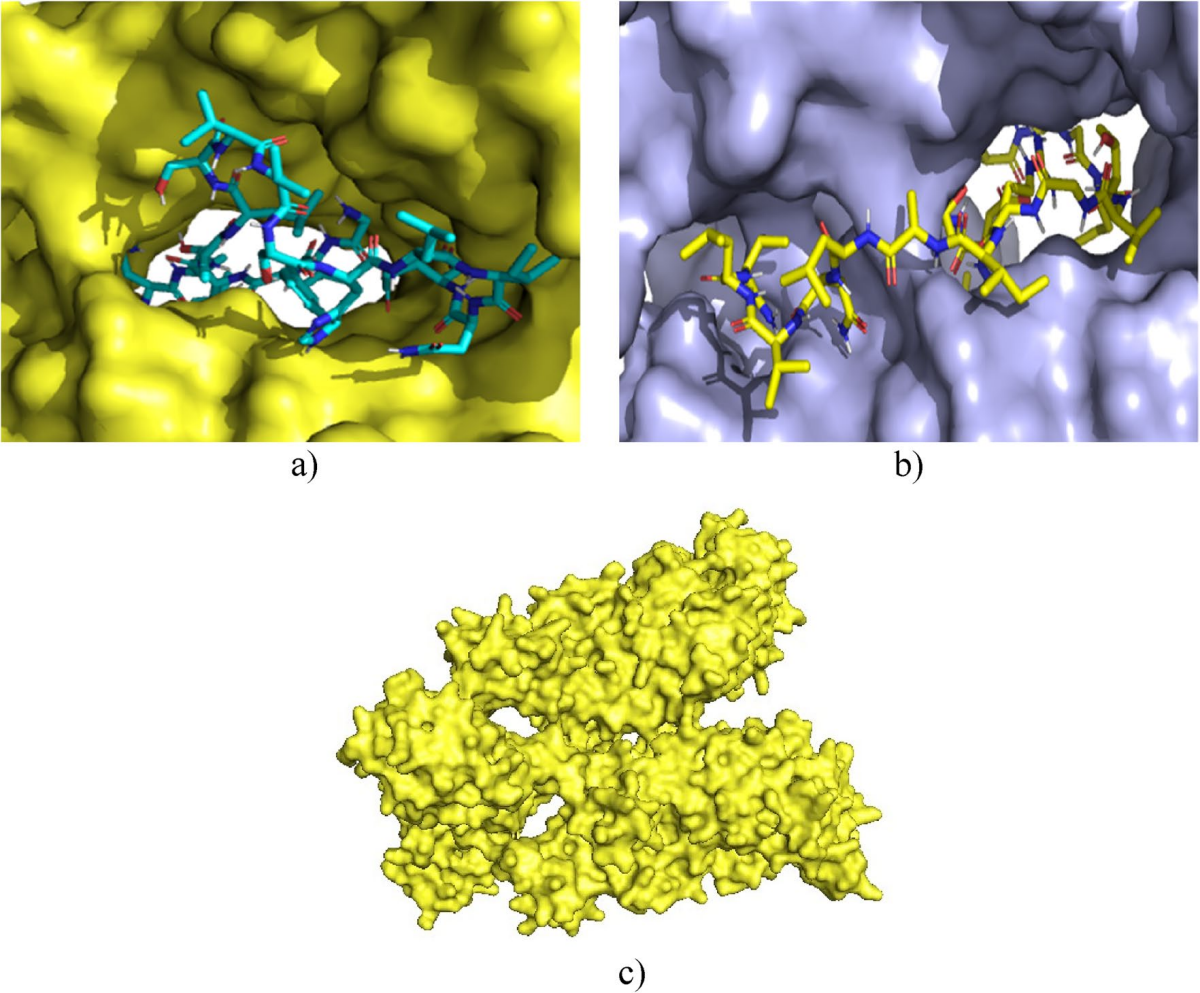
(**a**) Interaction of Cruzioseptin-4 (blue) with the enzyme Penicillin-binding protein g-Acyl 2A (yellow). (**b**) Interaction of Pictuseptin-1 (yellow) with the enzyme Penicillin-binding protein g-Acyl 2A (blue). (**c**) Enzyme Penicillin-binding protein g-Acyl 2A (yellow).

The efficacy of Penicillin g-Acyl 2A-binding protein inhibitors is due to their ability to form stable covalent complexes with their respective targets^57^. Ceftobiprole inhibitor is a new fifth-generation cephalosporin with antimicrobial activity^58^. It is known that in the type of enzyme-drug interactions, the size and charge parameters are essential to produce inhibition; this drug possesses a charge of -1 at pH 7, while the peptides studied possess a positive charge at this pH value^56^. The efficacy of this inhibitor consists of an excellent inhibitory interaction with the narrow active site groove of the enzyme, favouring its acylation and subsequent cell death^59^. However, unlike Cruzioseptin-4 and Pictuseptin-1, the size of the known inhibitor is smaller because it has 60 atoms and possesses a charge at pH 7 of -1. These differences prevent the studied peptides from fully interacting with the enzyme's active site, as illustrated in Fig. 3.

**Figure 4:**
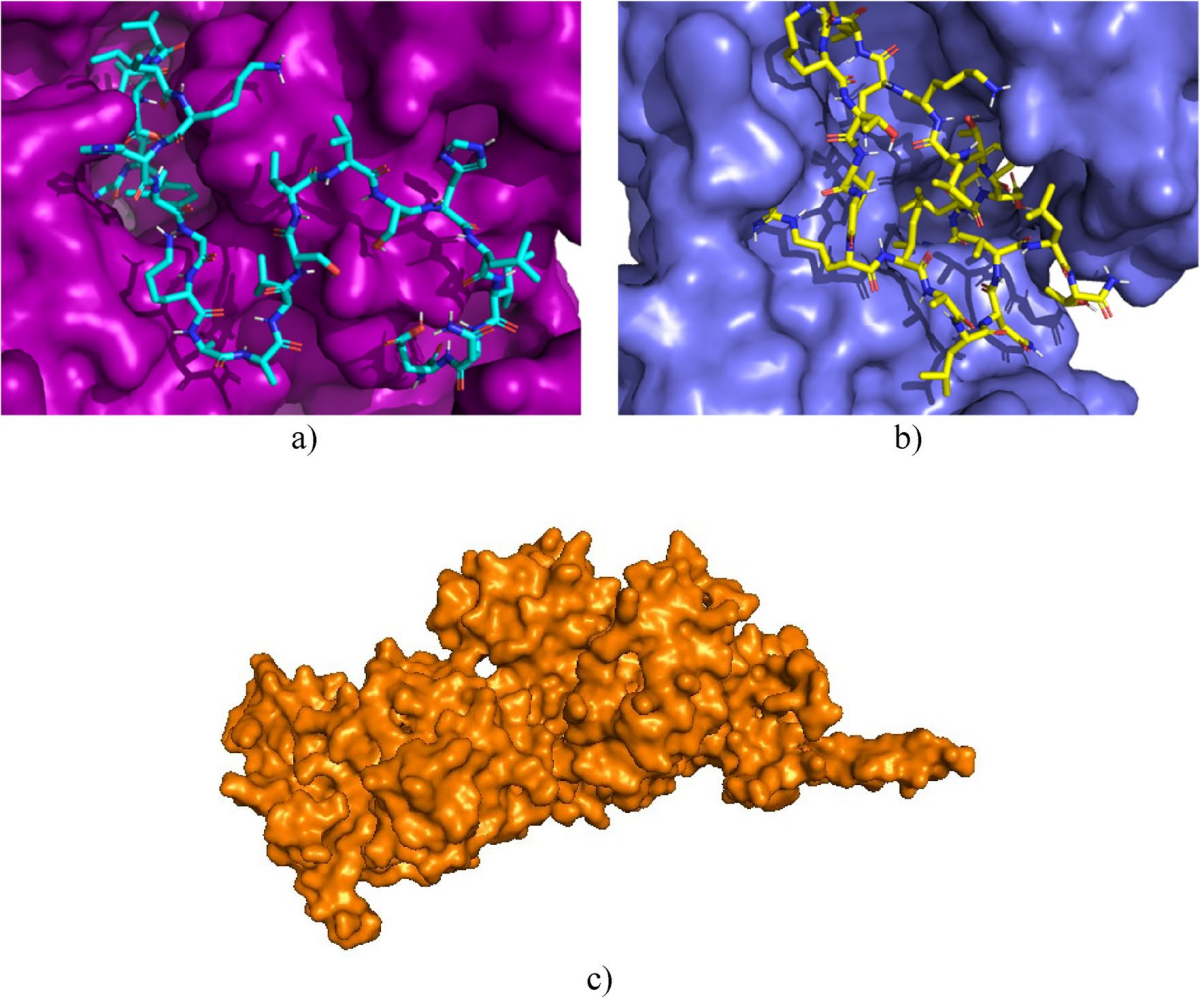
(**a**) Interaction of Cruzioseptin-4 (blue) with the enzyme Penicillin Transglycosidase Binding Protein 1b (purple). (**b**) Interaction of Pictuseptin-1 (yellow) with the enzyme Penicillin Transglycosidase Binding Protein 1b (blue). (**c**) Enzyme Penicillin Transglycosidase Binding Protein 1b (orange).

Penicillin-binding protein transglycosidase 1b is a bifunctional transglycosylase containing a transmembrane helix, two enzymatic domains, transpeptidase and transglycosylase; in addition, it has a domain composed of 100 amino acid residues with unknown functionalities and structures^60^. Its inhibitor, Moenomycin, is involved in bacterial cell wall synthesis, preventing the formation of the linear glycan strands of peptidoglycan^61^. Both the inhibitor and the peptides studied could bind to the active site since the active site has good availability, so the docking values are similar. In Fig. 4, the availability of the active site favours the interaction of the peptides with the enzyme.

**Figure 5:**
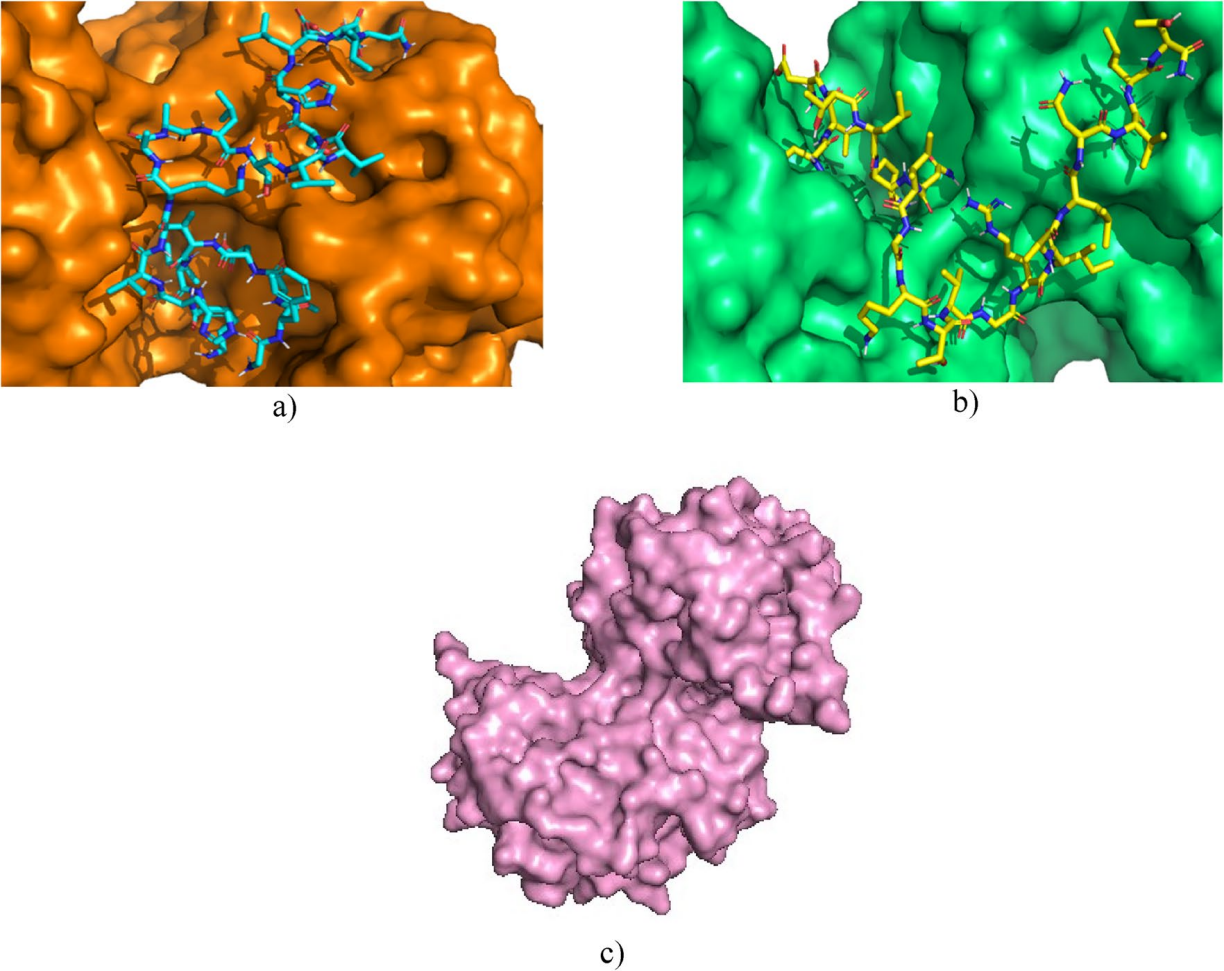
(**a**) Interaction of Cruzioseptin-4 (blue) with the enzyme AmiA hydrolase (orange). (**b**) Interaction of Pictuseptin-1 (yellow) with the enzyme AmiA hydrolase binding protein (green). (**c**) Enzyme AmiA hydrolase binding protein (pink).

In the case of the enzyme AmiA hydrolase, the values of the peptides studied do not differ much from the known inhibitor Muramil tetrapeptide; however, the three peptides are basic, but there is a difference in length, where the length of Pictuseptin-1 is 22, of Cruzioseptin-4 is 23 (Table 1) and of the known inhibitor is 4. This difference in size and the slight separation between the amino acids of the inhibitor facilitates its interaction with the enzyme, which is not the case for peptides^57,62^. In Fig. 5, the peptides have difficulty interacting with the active site due to the length of the chain.

**Figure 6:**
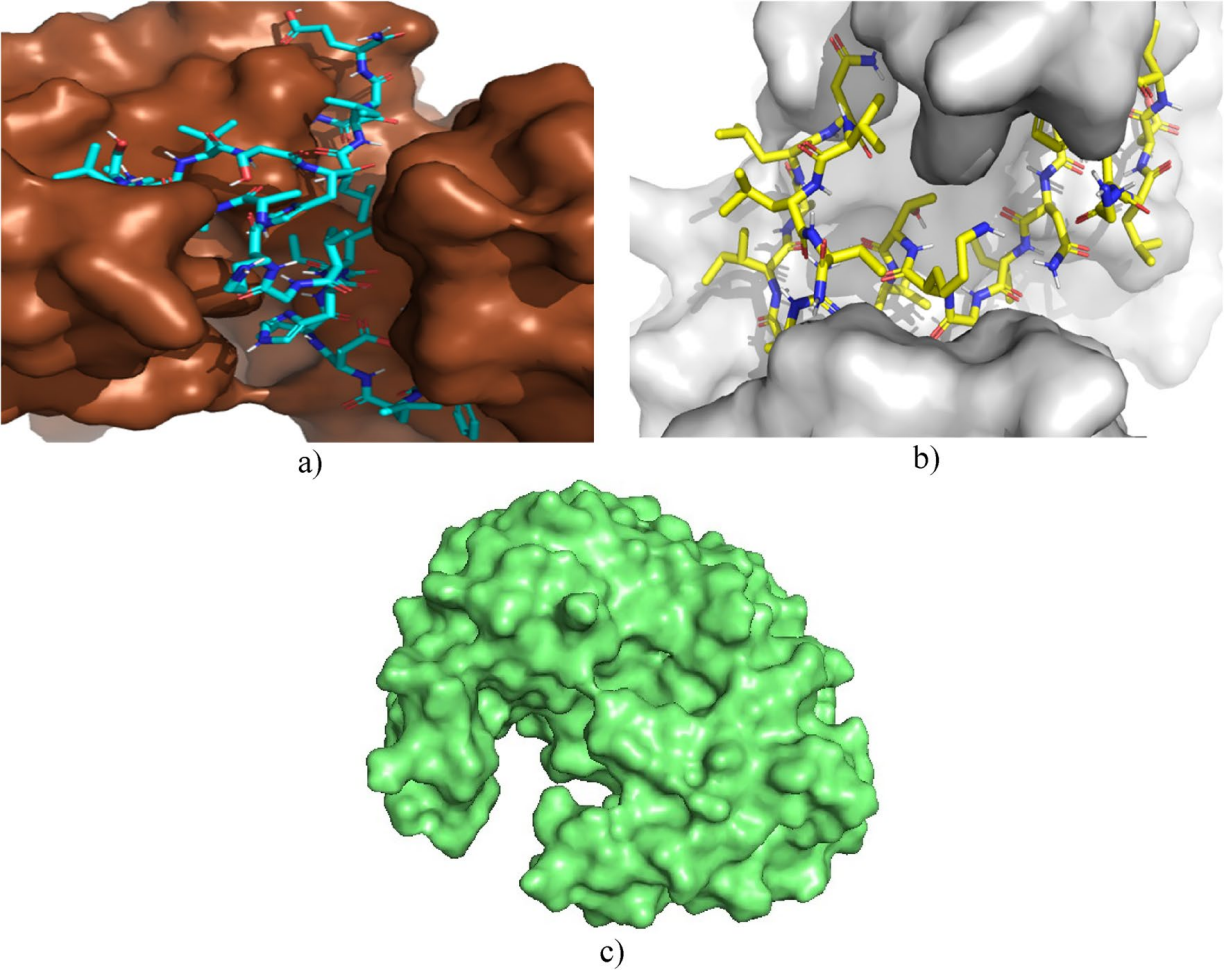
(**a**) Interaction of Cruzioseptin-4 (blue) with secreted aspartic protease enzyme (brown). (**b**) Interaction of Pictuseptin-1 (yellow) with secreted aspartic protease enzyme (grey). (**c**) Secreted aspartic protease enzyme (green).

The secreted aspartic protease enzyme has a structure with an 8-residue insertion and a short polar bond between two domains, where the inhibitor can bind in an extended conformation^63^. Proteases are characterised by breaking peptide bonds, so the docking values between the known inhibitor A70 and the peptides studied are not very distant^56^. Figure 6 shows that the peptides Cruzioseptin-4 and Pictuseptin-1 interact with the enzyme’s active site via an extended conformation.

In the case of the enzyme DNA gyrase B, the docking values between the known inhibitor ADP and the peptides studied are significant due to the difference in size and charge. The enzyme Exo-B-(1,3)-glucanase is known to possess hydrolase activity as a transferase and is characterised by having eight residues in its active site, of which 2 of them are glutamate residues acting as nucleophile and acid/base, respectively; in addition, there is an active site within a deep pocket; therefore, a suitable inhibitor should be based on the covalent binding mechanism and a transition state analogue^64^. Previous studies have shown that Castanospermine is a suitable inhibitor of lysosomal amyloglucosidases and glycosidases, also considering the number of atoms of this inhibitor is 28, which is lower compared to Cruzioseptin-4 and Pictuseptin-1, allowing a better interaction with the active site of this enzyme^65^.

On the other hand, Table 6 shows the docking values expressed in kcal/mol for Cruzioseptin-4 of the different molecules present in the bacterial cell membranes. The aim of this calculations is to get insights in which molecules inside the membrane may interact with the peptides helping them penetrate the cell membrane and cause the disruption proposed. Results show values range − 0.9 and − 2.1 kcal/mol. Likewise, the docking values expressed in kcal/mol of the different molecules with Pictuseptin-1 are shown, in which it can be observed that the range of values oscillates between − 0.6 and − 2.7 kcal/mol. These data indicate that the docking score is very low since the interaction is fragile, even though the mechanism is through electrostatic interactions.

**Table 6:** Docking values of Cruzioseptin-4 and Pictuseptin-1 for different molecules present in the cell membrane of *Staphylococcus aureus*, *Escherichia coli* and *Candida albicans.*

Cell membrane molecules affinity	Docking score (kcal/mol)	
	Cruzioseptin-4	Pictuseptin-1
Teichoic acid	− 2.1	− 1.2
1–3 glucan	− 1.2	− 0.6
Phosphatidylethanolamine	− 1.8	− 1.3
Glycophospholipids phosphomannolipid	− 1.2	− 2.7
Glycophospholipid GPLs	− 3.1	− 2.5
Lysyl-phosphatidylglycerol	− 1.4	− 2.6
Myristic acid	− 1.1	− 1.0
Oleic acid	− 1.1	− 1.2
Palmitic acid	− 0.9	− 1.7
Palmitoleic acid	− 1.7	− 1.4
Phosphatidylglycerol	− 1.4	− 1.3

The lipid composition of cytoplasmic membranes can vary, so it is an essential factor when analysing the docking values between different molecules; given that in most bacteria, the Phospholipid and Phosphatidylethanolamine (PE) predominates, it should be noted that there is a higher content of this PE phospholipid in Gram-negative bacteria than in Gram-positive bacteria. There are also some anionic lipids in bacterial membranes, but the most predominant is Phosphatidylglycerol and Cardiolipin; consequently, this would allow an excellent electrostatic interaction with the peptides because Cruzioseptin-4 and Pictuseptin-1 have positive charges (Table 1) and could generate a rupture of the cell wall^66^.

Table 7 shows the results of computational studies performed on Cruzioseptins-4, 2, 3, 16, 17 and Pictuseptin-1, which show antimicrobial effects against Gram-positive, Gram-negative and fungal pathogens. For the Penicillin-binding protein g-Acyl 2A and AmiA hydrolase enzymes, Pictuseptin-1 shows a lower docking score. For the enzyme DNA gyrase B and Exo-B-(1,3)-glucanase, the Cruzioseptin-3 peptide has the lowest docking score. For the enzyme Penicillin Binding Protein Transglycosidase 1b, Pictuseptin-1 and Cruzioseptin-3 have the same docking score of − 6.6 kcal/mol, being the most negative docking value. For the above Secreted aspartic protease, Cruzioseptin-16 presented the lowest docking value^20^.

**Table 7:** Docking score values between Cruzioseptins, Pictuseptin-1 and different enzymes.

Organism	Enzyme	Known inhibitor	Docking score (kcal/mol)					
			Pictuseptin-1	CZS-4	CZS-2**	CZS-3**	CZS-16**	CZS-17**
*S. aureus*	Penicillin-binding protein g-Acyl 2A	Ceftobiprole	− 7.1	− 6.2	− 6.1	− 5.8	− 6.0	− 5.8
	AmiA hydrolase	Muramyl tetrapeptide	− 6.2	− 6.0	− 5.4	− 4.8	− 5.4	− 5.6
*E. coli*	DNA gyrase B	ADP	− 5.7	− 5.6	− 6.3	− 6.8	− 6.1	− 4.0
	Penicillin Binding Protein Transglycosidase 1b	Moenomycin	− 6.6	− 6.1	− 6.4	− 6.6	− 6.0	− 6.3
*C. albicans*	Exo-B-(1,3)-glucanase	Castanospermine	− 5.9	− 5.1	− 6.5	− 6.8	− 5.6	− 5.4
	Secreted aspartic protease	A70*	− 5.5	− 6.3	− 6.2	− 6.5	− 7.0	− 6.6

*A70: N-etil-N-[(4-metilpiperazin-1-yl)carbonil]-D-fenilalanil-N-[(1S,2S,4R)-4-(butilcarbamoil)-1-(ciclohexilmetil)-2-hidroxi-5-metilhexil]-L-norleucinamida.

**Results taken from previous studies^17,20,46,47^.

All these results have shown that the most feasible mechanism of action for peptides belonging to the Cruzioseptin family is through cell membrane perturbation caused by electrostatic interactions occurring between the positively charged residues of the peptide and the phospholipids and negatively charged molecules of the cell membrane^20^.

The results of the computational studies on Cruzioseptin-4 and Pictuseptin-1 are similar to the ones found for other AMP extracted from frogs (Tables 3 and 7). In this sense, these new peptides are suggested to present antimicrobial activity and low haemolytic properties as the main function same as that of other cationic AMPs.

## Conclusion

Cruzioseptin-4 extracted from the frog *Cruziohyla calcarifer* and Pictuseptin-1 extracted from the frog *Boana Picturata* are cationic peptides that show antimicrobial activity against some pathogens *Staphylococcus aureus*, *Escherichia coli*, and the yeast *Candida albicans*. The physicochemical properties and computational modelling of Cruzioseptin-4 indicate a basic chain of 23 amino acids, a charge at pH 7 of + 1.2, a high percentage of hydrophobicity (52.17%) and an alpha-helical secondary structure. Similarly, Pictuseptin-1 has a basic chain of 22 amino acids, a charge at pH 7 of + 3, a high hydrophobicity of 45.45% and a predominant alpha-helical secondary structure. Molecular docking studies for the two peptides show that an enzymatic inhibition mechanism is not feasible due to their size and charges, as these factors hindered the binding of these inhibitors with the different active sites studied. On the other hand, a viable mechanism would be an attack on the bacterial cell membrane, which would cause cell lysis, a product of the electrostatic interactions of the positive regions of the peptides with different components of the cell membranes of negative charges.

## Data Availability

The datasets generated during and/or analysed during the current study are available from the corresponding author on reasonable request.
